# Delusion of pregnancy - what the literature says?

**DOI:** 10.1192/j.eurpsy.2024.1570

**Published:** 2024-08-27

**Authors:** I. M. Figueiredo

**Affiliations:** Clinic 3, Psychiatric Hospital Center of Lisbon, Lisbon, Portugal

## Abstract

**Introduction:**

Delusion of pregnancy (DP) is a false and persistent belief of being pregnant despite realistic evidence to the contrary. Being considered a rare phenomenon, more cases of DP have been reported lately, however the literature about this topic is still scarce.

**Objectives:**

Clarify the etiology and clinical aspects of this pathology in order to diagnose and to treat it properly.

**Methods:**

A search on Pubmed was performed using the MeSH terms “delusion pregnancy” or “pseudocyesis”. The DSM-5 and ICD-10 were also a source of information.

**Results:**

DP can be sometimes confused with other disorders, like pseudocyesis, pseudo-pregnancy and Couvade syndrome, but it is important to differentiate all of them to have a clear view of the pathology and follow a correct approach to the problem.

DP can manifest isolatedly, but it is more commonly associated with other diseases. Etiologically, several factors can intercede: biological, psychosocial and cultural factors, iatrogenic factors and coenaesthesis processes.

Demographically, about 50% of the patients are 20-40 years old and the most common psychiatric diagnoses are schizophrenia, bipolar disorder and depression.

Concerning the treatment, it is essential to exclude non-psychiatric causes and treat those, if present. After doing so, the therapeutical approach can be non-pharmacological, using psychotherapy or electroconvulsive therapy, although the latter has inconclusive results and sometimes it only remits the comorbid depressive symptoms; or pharmacological using 2nd generation antipsychotics.

In general, there is now a good response in 50-64% of the cases (Bera and Sakar, *Indian J Psychol Med* 2015;37(2)131-137) (Yadov *et al*, *Indian J Psychol Med* 2012;34(1) 82-84).

**Conclusions:**

DP can be a psychiatric diagnosis itself or a manifestation of other psychiatric or non-psychiatric disorder so we must be allert to make a precise differential diagnosis. Its genesis is multifactorial and that must be taken into account when thinking about its treatment approach.

In the past, the prognosis of the DP wasn’t good, but in the recent literature it was found a good response in more than half of the patients treated accordingly.

**Disclosure of Interest:**

None Declared

